# Continuous versus intermittent temperature measurement in the detection of fever in critically ill patients

**DOI:** 10.1186/cc14084

**Published:** 2015-03-16

**Authors:** A Heyneman, V Bosschem, N Mauws, D Van Der Jeught, E Hoste, J Decruyenaere, J De Waele

**Affiliations:** 1 Ghent University Hospital, Ghent, Belgium

## Introduction

An elevated body temperature is one of the four criteria in diagnosing systemic inflammatory response syndrome (SIRS), and is often used at the bedside to trigger diagnostic investigations for infection. Standard intermittent temperature measurement may, however, delay the detection of an elevated temperature or miss this altogether. The aim of the study is to compare intermittent noninvasive versus continuous invasive body temperature measurement as a tool to detect an elevated body temperature.

## Methods

This was a secondary analysis of a prospective study in 25 critically ill patients comparing different measurement techniques. Temperature was measured intermittently with an axillary digital thermometer every 4 hours, and a urinary bladder thermistor catheter was used for continuous temperature measurement; the latter was considered the reference method. Fever (core temperature ≥38.3°C) episodes occurring within 60 minutes after each other were classified as one episode. We compared the fever detection rate of both Methods and calculated the difference in timing between both techniques.

## Results

Twenty-nine episodes of fever were detected in 10 patients (seven male) with a median APACHE II score of 10 (IQR 3 to 24) and a median SOFA score of 10 (IQR 8 to 11). Median duration of a fever episode was 1 hour 24 minutes (IQR 47 minutes to 2 hours 59 minutes) and median maximum temperature was 38.4°C (IQR 38.3 to 38.7). Median axillary temperature was 0.7°C (IQR 0.3 to 0.9) lower than core temperature. Using intermittent, non-invasive measurement, 27 out of 29 fever episodes (93.1%) remained undetected.

## Conclusion

Intermittent, non-invasive temperature measurement failed to detect most of the fever episodes as measured by a bladder thermistor catheter and should not be used to screen for elevated body temperature in critically ill patients.

